# Oncolytic H-1 Parvovirus Shows Safety and Signs of Immunogenic Activity in a First Phase I/IIa Glioblastoma Trial

**DOI:** 10.1016/j.ymthe.2017.08.016

**Published:** 2017-08-24

**Authors:** Karsten Geletneky, Jacek Hajda, Assia L. Angelova, Barbara Leuchs, David Capper, Andreas J. Bartsch, Jan-Oliver Neumann, Tilman Schöning, Johannes Hüsing, Birgit Beelte, Irina Kiprianova, Mandy Roscher, Rauf Bhat, Andreas von Deimling, Wolfgang Brück, Alexandra Just, Veronika Frehtman, Stephanie Löbhard, Elena Terletskaia-Ladwig, Jeremy Fry, Karin Jochims, Volker Daniel, Ottheinz Krebs, Michael Dahm, Bernard Huber, Andreas Unterberg, Jean Rommelaere

**Affiliations:** 1 Department of Neurosurgery, University Hospital, Im Neuenheimer Feld 400, 69120 Heidelberg, Germany; 2 Coordination Centre for Clinical Trials, University Hospital, Marsilius-Arkaden, Tower West, Im Neuenheimer Feld 130.3, 69120 Heidelberg, Germany; 3 Department of Tumor Virology, German Cancer Research Center (DKFZ), Im Neuenheimer Feld 242, 69120 Heidelberg, Germany; 4 Department of Neuropathology, University Hospital, Im Neuenheimer Feld 220, 69120 Heidelberg, Germany; 5 Clinical Cooperation Unit Neuropathology, German Cancer Consortium (DKTK), German Cancer Research Center (DKFZ), Heidelberg, Germany; 6 Department of Neuroradiology, University Hospital, Im Neuenheimer Feld 400, 69120 Heidelberg, Germany; 7 University Hospital Pharmacy, Im Neuenheimer Feld 670, 69120 Heidelberg, Germany; 8 Department of Neuropathology, University Medical Center, Georg August University, 37099 Göttingen, Germany; 9 Eurofins BioPharma Product Testing, Behringstraße 6/8, 82152 Planegg, Germany; 10 Laboratory Prof. Dr. Gisela Enders & Colleagues, MVZ and Institute of Virology, Infectious Diseases and Epidemiology e.V., Stuttgart, Germany; 11 ProImmune, The Magdalen Centre, Oxford Science Park, Oxford OX4 4GA, UK; 12 IASON Consulting, Mühlenstraße 26A, 52382 Niederzier, Germany; 13 Department of Transplantation Immunology, Institute of Immunology, University Hospital, Im Neuenheimer Feld 305, 69120 Heidelberg, Germany; 14 Oryx GmbH & Co. KG, Marktplatz 1, 85598 Baldham, Germany

**Keywords:** oncolytic parvovirus, glioblastoma, clinical trial, tumor microenvironment

## Abstract

Oncolytic virotherapy may be a means of improving the dismal prognosis of malignant brain tumors. The rat H-1 parvovirus (H-1PV) suppresses tumors in preclinical glioma models, through both direct oncolysis and stimulation of anticancer immune responses. This was the basis of ParvOryx01, the first phase I/IIa clinical trial of an oncolytic parvovirus in recurrent glioblastoma patients. H-1PV (escalating dose) was administered via intratumoral or intravenous injection. Tumors were resected 9 days after treatment, and virus was re-administered around the resection cavity. Primary endpoints were safety and tolerability, virus distribution, and maximum tolerated dose (MTD). Progression-free and overall survival and levels of viral and immunological markers in the tumor and peripheral blood were also investigated. H-1PV treatment was safe and well tolerated, and no MTD was reached. The virus could cross the blood-brain/tumor barrier and spread widely through the tumor. It showed favorable pharmacokinetics, induced antibody formation in a dose-dependent manner, and triggered specific T cell responses. Markers of virus replication, microglia/macrophage activation, and cytotoxic T cell infiltration were detected in infected tumors, suggesting that H-1PV may trigger an immunogenic stimulus. Median survival was extended in comparison with recent meta-analyses. Altogether, ParvOryx01 results provide an impetus for further H-1PV clinical development.

## Teaser

Through an early-phase clinical trial in recurrent glioblastoma patients, Geletneky et al. show that the oncolytic H-1 parvovirus is safe, well tolerated, and able to establish an immunogenic tumor microenvironment. Blood-brain/tumor crossing and favorable survival compared with historical controls make this virus an interesting candidate for further clinical development.

## Introduction

Glioblastoma is the most aggressive primary human brain tumor. Currently, median survival is in the range of only 13–15 months at first diagnosis^1^ and 6–9 months at recurrence.^2^ Improved treatments are thus urgently needed.

One novel approach, oncolytic virotherapy, exploits the ability of replicating oncolytic viruses (OVs) to selectively kill tumor cells,^3^ as demonstrated in both preclinical settings and various clinical trials.^4^ Mounting evidence shows that OV infection can also induce specific antitumor immune effects, both through the production or release (upon cell lysis) of neo-antigens and via a virus-triggered immunogenic process causing tumor cell death.^5^ The virus inoculum can thus act as an oncolytic vaccine, and concepts for combining OV infection with current immunotherapies such as checkpoint inhibition are under investigation.^6^

Initial oncolytic virotherapy trials in glioblastoma were performed with herpes simplex virus,^7^, ^8^, ^9^, ^10^ adenovirus,^11^ or reovirus^12^, ^13^ injected either directly into the tumor or into the adjacent brain. They demonstrated the safety of this approach, but no clinical efficacy. Recently a second wave of trials has been completed (but not yet reported). An extended phase I trial using a replicating retrovirus harboring a prodrug-converting enzyme has yielded promising results.^14^

Here, we report on the first use of oncolytic H-1 parvovirus (H-1PV), a small, non-enveloped, single-stranded DNA virus^15^ whose natural host is the rat,^16^ in patients with recurrent glioblastoma. Humans are not naturally infected and therefore lack neutralizing antibodies.^17^ Two previous applications of H-1PV in humans revealed no virus-related pathogenic effects.^18^, ^19^ The oncosuppressive activity of H-1PV was demonstrated in numerous preclinical studies in glioblastoma and other tumor models.^20^, ^21^ In rats, H-1PV can cross the blood-brain barrier, causing intracranial tumor regression after intravenous injection.^22^ Tumor cells are vulnerable to the direct cytotoxic action of H-1PV because they contain higher levels than normal cells of multiple determinants essential to the regulation of the oncotoxic H-1PV protein NS1 (cellular replication and transcription factors, components of metabolic pathways).^23^ In animal models, cellular immune responses have been found to potentiate the oncosuppressive effect of H-1PV.^20^

ParvOryx01, the first dose-escalating clinical trial of H-1PV (pharmaceutical formulation: ParvOryx) in patients with malignant brain tumors, investigated local and systemic H-1PV treatment in glioblastoma patients. The primary objectives were to determine safety and tolerability, virus pharmacokinetics, shedding, and a maximum tolerated dose (MTD). Evidence of antitumor activity was assessed by progression-free survival (PFS) and overall survival (OS) and by histological, immunological, and virological changes in tumor specimens. In contrast with most previous trials, the ParvOryx01 design^24^ provided for the investigation of tumor tissue after treatment, a prerequisite to gaining in-depth understanding of the mode of action of the agent used and to devising possible improvements.

## Results

### Patients and Treatment

**Figure 1:**
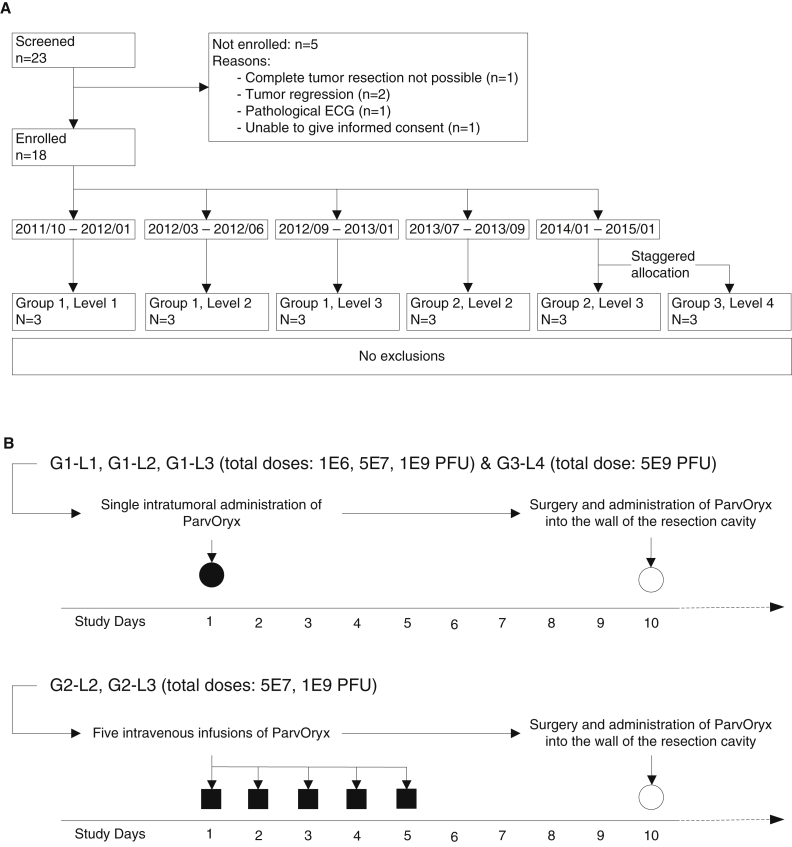
Schedule of ParvOryx Administration and Flow Chart of the Trial (A) Flow chart of the trial according to the CONSORT statement. The time interval assigned to each group and dose level represents the calendar period of patient enrollment into the corresponding cohort. (B) Schematic representation of the schedule of ParvOryx administration. Upper panel: treatment in G1 and G3. Intratumoral administration was performed through an intracranial catheter over approximately 30 min. Lower panel: treatment in G2. All five administrations were given as 2 hr intravenous infusions. In all groups on day 10, the remaining 50% of the total ParvOryx dose was injected into the walls of the resection cavity at multiple locations. PFU, plaque-forming units.

Eighteen patients (mean age: 57.8 ± 10.6 years) with a history of one previous glioblastoma resection and subsequent radiotherapy were enrolled in ParvOryx01 (Figure 1A; Table 1). Key eligibility criteria were: age ≥18 years; solid, non-metastatic, progressive primary or recurrent glioblastoma scheduled for complete or subtotal resection; life expectancy ≥3 months; Karnofsky performance score ≥60; and avoidance of exposure to immunocompromised individuals and infants ≤18 months of age for 28 days after the first ParvOryx dose. Treatment with anti-angiogenic substances within 21 days, radiotherapy within 90 days, and chemotherapy within 4 weeks prior to study inclusion were not allowed. Fifteen patients had received concomitant temozolomide (TMZ) as first-line therapy, whereas three had instead been treated with bevacizumab and irinotecan.^25^ MGMT (O^6^-methylguanine-DNA methyltransferase) promoter methylation was present in two patients, and all were isocitrate dehydrogenase 1 (IDH1) mutation-negative. Most patients had no or few symptoms, as assessed by Karnofsky status. Tumor size, defined as the maximal cross-sectional area of contrast enhancement on axial MRI planes, differed substantially between individual patients, but subtotal to total resection was achieved in all patients.

**Table 1:** Patient Characteristics at Study Entry

Subject ID	Age (years)	Sex	Treatment Group	Dose (PFU)	Previous Therapies	MGMT Methylation	IDH1 Mutation	Cross-Sectional Area (mm^2^)	KPS
1-01	51	male	G1-L1	1E6	S, RAD, TMZ	ND	neg	112	100
1-02	42	male	G1-L1	1E6	S, RAD, TMZ	no	neg	108	80
1-03	62	male	G1-L1	1E6	S, RAD, TMZ	no	neg	266	100
2-04	70	male	G1-L2	5E7	S, RAD, TMZ	NA	neg	288	100
2-05	53	female	G1-L2	5E7	S, RAD, TMZ	no	neg	3,300	100
2-06	64	male	G1-L2	5E7	S, RAD, TMZ	no	neg	2,772	80
3-07	48	female	G1-L3	1E9	S, RAD, TMZ	no	neg	805	80
3-08	44	male	G1-L3	1E9	S, RAD, BEV, IRI	no	neg	731	100
3-09	45	male	G1-L3	1E9	S, RAD, BEV, IRI	no	neg	638	70
4-10	69	male	G2-L2	5E7	S, RAD, TMZ	no	neg	1,925	60
4-11	47	male	G2-L2	5E7	S, RAD, BEV, IRI	no	neg	629	100
4-12	64	male	G2-L2	5E7	S, RAD, TMZ	NA	NA	770	90
5-13	66	male	G2-L3	1E9	S, RAD, TMZ	ND	neg	1,519	90
5-14	52	male	G2-L3	1E9	S, RAD, TMZ	yes	neg	336	90
5-15	55	female	G2-L3	1E9	S, RAD, TMZ	no	neg	1,056	100
6-16	62	female	G3-L4	5E9	S, RAD, TMZ	no	neg	575	90
6-17	76	male	G3-L4	5E9	S, RAD, TMZ	yes	neg	2,184	100
6-18	71	male	G3-L4	5E9	S, RAD, TMZ	no	neg	1,881	90

BEV, bevacizumab; IDH1, isocitrate dehydrogenase 1; IRI, irinotecan; KPS, Karnofsky performance status; MGMT, O^6^-methylguanine-DNA methyltransferase; NA, not available; ND, not determinable; neg, negative; PFU, plaque-forming units; RAD, radiation therapy; S, surgery; TMZ, temozolomide.

The 18 patients were assigned to two treatment arms differing in the mode of first virus application. In arm 1, comprising groups 1 and 3 (G1 and G3), the first dose of ParvOryx was injected intratumorally. In arm 2, containing G2, the patients initially received five intravenous virus infusions on days 1–5. On day 10, all patients of both arms underwent tumor resection, and virus was re-injected around the resection cavity (Figure 1B).

### Treatment Tolerance

Whatever the administration route, ParvOryx treatment showed no dose-dependent side effects or dose-limiting toxicity (DLT). It had no impact on safety laboratory parameters, except an isolated, slight-to-moderate increase in C-reactive protein without clinical symptoms, observed 3 days after intratumoral injection in all three G3-L4 patients. No changes in electrocardiogram or vital signs were observed. All but one intercurrent adverse event (AE) were related to the underlying disease or its complications and were unrelated to ParvOryx. Twelve AEs were classified as “serious,” i.e., required hospitalization or were life-threatening or otherwise medically relevant (Tables S1 and S2). The only event possibly caused by ParvOryx was observed in patient 6-16 (G3-L4), meeting the criteria of a suspected unexpected serious adverse reaction (SUSAR). This first patient in the highest dose subgroup showed a progressively deteriorating level of consciousness starting on treatment day 12, 2 days after intracerebral administration of ParvOryx. The postoperative computed tomography (CT) scan was consistent with new signs of hydrocephalus requiring surgical interventions. During reoperation, however, no elevated intracranial pressure was observed. Cerebrospinal fluid (CSF) analysis showed high protein and lactate levels, but no elevated cell counts. No infectious virus particles were found in the CSF at any time. On the basis of laboratory and auxiliary analyses (electroencephalogram, MRI), one could exclude active inflammation (e.g., encephalitis, meningitis), metabolic deterioration, and seizures, and no direct ParvOryx-related cause could be established. During the evaluation of the event, the sponsor temporarily and voluntarily suspended recruitment for the trial. The patient never regained consciousness and, after 6 months, life support was suspended on request of the family. After thorough discussion of the case with the Data Safety Monitoring Board (DSMB) and the German regulatory authority (Paul-Ehrlich-Institut [PEI], Langen, Germany), the event was not considered as a DLT due to unproven causality, and the trial was continued as planned. The next two G3-L4 patients showed no side effects possibly related to ParvOryx.

### Clinical Outcome

The information on individual clinical responses is given in Table 2 and Figure S1. Overall, during the regular trial follow-up (up to 6 months post-enrollment), 12 patients showed progressive disease or died. PFS at 6 months was 27%, and median PFS was 111 days. Five patients died during follow-up of 6 months. OS was approximately 72%, and median OS was 464 days. Because fewer than 9 patients died by 6 months, the calculation of the median OS was based on survival data for all 18 patients obtained through continuing visits to the trial center or telephone interviews. PFS and OS were independent of ParvOryx dose or administration route.

**Table 2:** Individual Clinical Responses in All 18 Patients

Treatment Group	Subject ID	Progression-free Survival (PFS)^a^		Overall Survival (OS)^b^	
		Days^c^	Direct Documentation^d^	Days^c^	Direct Documentation^d^
G1-L1	1-01	171	no	822	yes
	1-02	18	yes	464	yes
	1-03	170	no	770	yes
G1-L2	2-04	161	no	1226	yes
	2-05	19	yes	357	yes
	2-06	15	yes	151	yes
G1-L3	3-07	111	yes	503	yes
	3-08	119	yes	492	yes
	3-09	53	yes	337	yes
G2-L2	4-10	55	no	97	yes
	4-11	28	yes	181	yes
	4-12	169	no	220	no
G2-L3	5-13	17	yes	543	no
	5-14	111	yes	507	no
	5-15	112	yes	196	no
G3-L4	6-16	46	no	184	yes
	6-17	56	yes	153	yes
	6-18	19	yes	101	yes

a According to the trial protocol, the study visits could take place within a 2-week interval before or after the respective dates. Therefore, the values of the individual PFS may slightly vary from the predetermined ones.

b Whenever applicable, patients were followed up for OS beyond the regular study follow-up period of 6 months by means of telephone interviews or visits to the trial center. Therefore, the timing of actual censoring for individual OS may exceed 6 months.

c PFS, days after surgery; OS, days after first administration of ParvOryx.

d PFS, progressive disease documented by trial-specific investigations (MR scans) versus third-party communication; OS, date of death known versus censoring at end of the study.

### Pharmacokinetics

**Figure 2:**
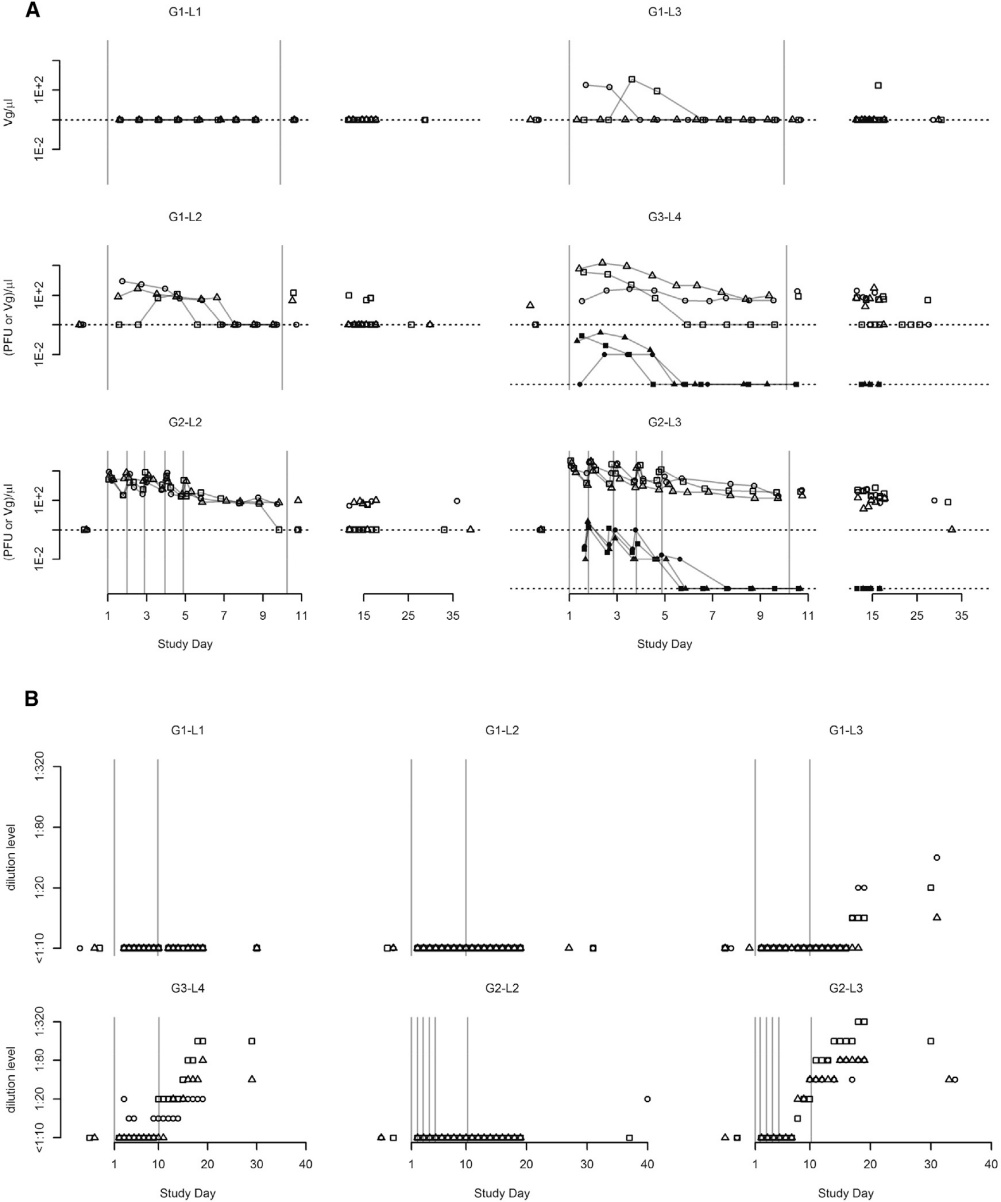
Pharmacokinetics and Seroconversion (A) Concentration over time, by cohort, of virus genomes (Vg; outline symbols) and infectious particles (PFU; solid symbols) in blood. Values below lower limits of quantification (LLOQ) are denoted by dotted lines. (B) Time course of anti-drug antibodies (ADAs) by cohort, as detected in a hemagglutination inhibition test.

Blood concentrations of H-1PV viral genomes (Vg) and infectious particles were measured to determine systemic virus availability. After intratumoral administration, both Vg and infectious virus particles appeared in the blood of eight of the nine patients in the subgroups G1-L2, G1-L3, and G3-L4, whereas no Vg were detected in the blood of any G1-L1 patient, indicating that H-1PV can cross the blood-brain/tumor barrier in a dose-dependent manner also in humans (Figure 2A, upper panels). After intravenous administration, blood Vg concentrations continuously rose during each infusion period. Dose sub-proportionality of systemic exposure was observed within the investigated dose range, the ratio of maximum concentrations being about one order of magnitude. This makes virus pharmacokinetics reliably predictable. After each post-infusion peak, Vg concentrations dropped rapidly, by approximately two orders of magnitude over 22 hr (Figure 2A, lower panels). This was most likely due to broad distribution of the virus to non-target body organs, in keeping with preclinical data showing highest concentrations in the liver and spleen.^26^ In all six intravenous patients, low Vg blood levels were detectable until day 10, when ParvOryx was re-injected intracerebrally after (sub)total tumor removal. Dose-dependent crossing of the blood-brain/tumor barrier was again observed in all patients after multifocal injection around the resection cavity (at 30–60 sites, depending on cavity size) following tumor resection.

### H-1PV-Specific Antibody Formation

A virus dose-dependent anti-H-1PV seroconversion was observed. Although no H-1PV-specific antibodies were detected by hemagglutination inhibition (HI) assay in any patient of G1-L1, G1-L2, and G2-L2 between days 1 and 30, all G1-L3, G3-L4, and G2-L3 patients showed seroconversion (Figure 2B). Higher doses led to earlier antibody appearance and to higher antibody titers (G3-L4 versus G1-L3). At the same total virus dose of 1E9 plaque-forming units (PFU), intravenously treated patients showed earlier and stronger seroconversion compared with intratumorally treated patients (G2-L3 versus G1-L3). In an infectivity assay, the antibodies displayed neutralizing capacity (data not shown).

### Virus Transmission from Study Patients to Third Persons: Risk Assessment

Samples of feces, saliva, and urine were tested for the presence of Vg and, when positive, infectious virus. As expected from preclinical data in rodents,^27^ Vg were excreted primarily via the feces, and concentrations were dose dependent (maximum: 376 Vg/mg in one G3-L4 patient). In intratumorally treated patients, fecal H-1PV excretion was detected only at the highest virus dose (G3-L4), whereas all but one intravenously treated patient tested positive at lower doses (G2-L2 and G2-L3). No patient had detectable Vg in feces beyond day 20. In urine, Vg were detected only in G2-L3 patients, yet at low concentrations (maximum: 11 Vg/μL) and not beyond day 11. All saliva samples tested negative. Importantly, no infectious virus particles were detected in any feces or urine sample with a Vg level above the lower limits of quantification (LLOQ) (data not shown). This rules out the risk of virus transmission from study patients in the administered dose range.

### H-1PV Expression in Tumor Tissue

**Figure 3:**
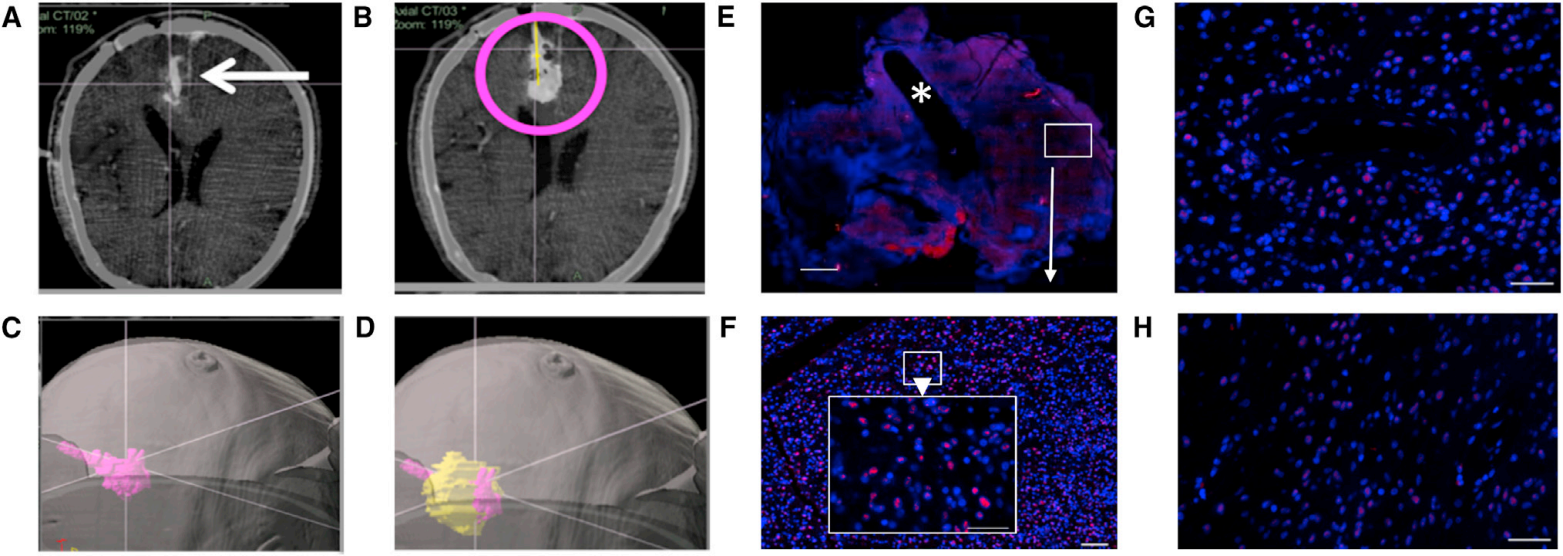
Intratumoral Virus Distribution and Ability to Cross the Blood-Brain/Tumor Barrier (A–D) Distribution of the H-1PV inoculum after intratumoral injection (CT scan, patient 3-08). (A) Verification of correct catheter placement in a left occipital tumor by intraoperative CT prior to injection. (B) CT scan after injection of 1 mL of virus inoculum (magenta circle). (C) Three-dimensional segmenting of virus inoculum. (D) Overlay of reconstructed tumor (yellow) with virus inoculum (magenta), showing very little virus signal outside the tumor margins. (E and F) Virus distribution after intratumoral injection (patient 3-09). (E) FISH staining against H-1PV RNA of en bloc resected tumor with visible catheter track (asterisk). Scale bar, 2,000 μm. An area distant from the catheter track (white box) is magnified in (F) (white arrow). (F) Higher magnification (scale bar of whole image, 50 μm; scale bar of zoomed area, 100 μm) showing a strong hybridization signal for H-1PV RNA (red) at a distance of 7,000 μm from the catheter, thereby proving wide virus distribution through the tumor after local injection. (G and H) Intratumoral detection of H-1PV transcripts by FISH after intravenous injection (patient 4-10) indicating crossing of the blood-brain/tumor barrier. Hybridization signals are detected both around intratumoral blood vessels (G) and in blood vessel distant tumor areas (H). Scale bars, 50 μm.

Because the virus was suspended in Ringer solution with 48% iodixanol (an X-ray contrast agent), it was possible to visualize the initial distribution of ParvOryx after local injection by CT performed within 30 min post-surgery (Figures 3A–3D). The observed distribution proved that slow injection (1 mL in 30 min) kept ParvOryx mainly in the tumor tissue, and that no virus dose was lost by backflow along the catheter, a common problem of local injections in the brain.

**Figure 4:**
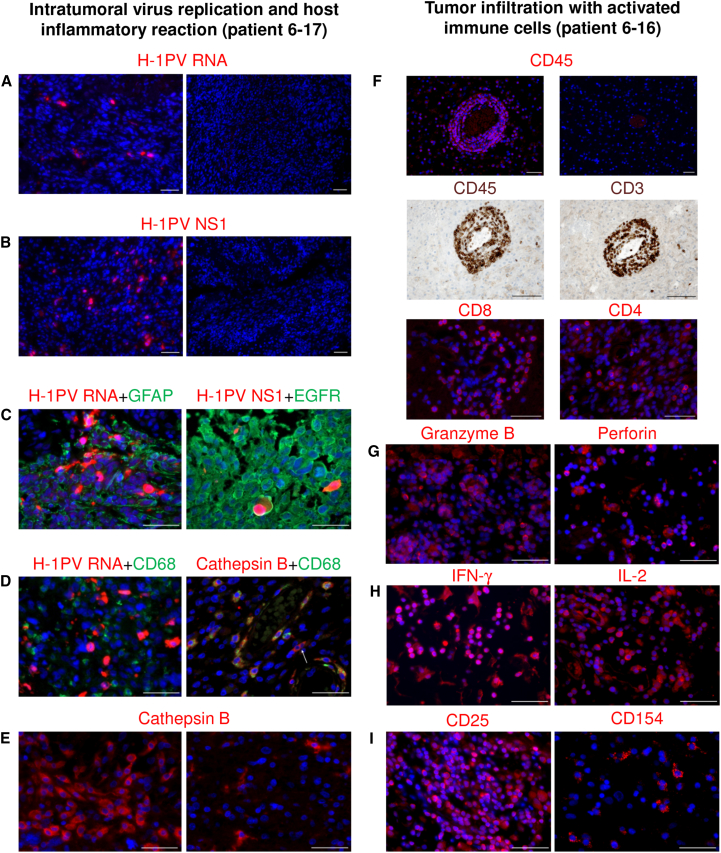
In Situ Analysis of Tumors Resected after Local ParvOryx Administration (A–E) Intratumoral virus replication and host inflammatory reaction (patient 6-17). (A and B) H-1PV transcripts (A) and NS1 proteins (B) were detected in virus-injected tumor tissue (left), but not in historical controls (right). (C) Double staining was performed for (left) viral RNA (red) and glial fibrillary acidic protein (green), or (right) viral NS1 (red) and epidermal growth factor receptor (green). (D) H-1PV-transcript-accumulating tumor cells (red) stained negative for the macrophage marker CD68 (green) (left). In contrast, the majority of cathepsin B (CTSB)-positive cells (red) expressed CD68 (green) (right). CTSB^+^/CD68^−^ cells were also detected (arrow). (E) Increased CTSB expression was observed in ParvOryx-treated tumor (left), as compared with historical control (right). (F–I) Tumor infiltration with activated immune cells (patient 6-16). (F) Upper two panels: the treated tumor showed increased leukocytic (CD45^+^) infiltration (left) compared with historical control (right). Middle two panels: tumor infiltrates (CD45, left) consisted predominantly of CD3^+^ T lymphocytes (right). Lower two panels: the T cell population included both CD8^+^ (left) and CD4^+^ (right) lymphocytes. (G–I) Several markers of immune cell activation were also detected in the ParvOryx-treated tumor: (G) granzyme B (left) and perforin (right), (H) IFN-γ (left) and IL-2 (right), and (I) CD25 (left) and CD154 (CD40L) (right). Scale bars, 50 μm.

H-1PV presence and distribution in resected tumor tissue were determined by fluorescence in situ hybridization (FISH). Viral DNA was revealed in 11 out of 12 tumors of intratumorally treated patients (Table 3). This was confirmed by quantitative real-time PCR and detection of infectious virus particles. Virions originating from three resected tumors (patients 3-07, 3-08, and 3-09) were analyzed for their genomic integrity. No mutations were detected compared to the input virus DNA (GenBank: JX505432.1) by sequencing the entire Vg. Tumors of all (except two G1-L1) patients displayed ParvOryx dose-dependent positivity for H-1PV transcripts (Figure 4A; Table 3). Positive FISH signals were not confined to the virus inoculation site but appeared also in catheter-distant tumor areas, confirming that local ParvOryx injection can lead to meaningful penetration into tumor tissue (Figures 3E and 3F). The presence of H-1PV RNA correlated with that of the cytotoxic viral protein NS1 (Figure 4B; Table 3). NS1-positive cells were found clustered within regions of histologically confirmed solid tumor tissue (Figures S2A and S2B). Cells accumulating H-1PV transcripts and NS1 were found mainly in areas staining positive for glial fibrillary acidic protein (GFAP) and epidermal growth factor receptor (EGFR) expression, suggesting virus replication in tumor cells (Figure 4C). To assess possible virus replication in tumor-associated microglia/macrophages (TAM), we performed CD68-FISH staining. Although some low-level virus transcription was observed in a minor TAM fraction, high H-1PV transcript levels (scored as +++; Table 3) were detected exclusively in non-macrophage cells (Figure 4D, left). This parallels previous findings that stimulated human peripheral blood mononuclear cells (PBMCs) support only abortive H-1PV infection.^28^ After intravenous ParvOryx administration, H-1PV RNA was revealed in four out of six resected tumors (Table 3; Figures 3G and 3H). Viral DNA was detected in three out of six tumors (Table 3). These observations were confirmed by quantitative real-time PCR. Together with the results of preclinical virus distribution studies in rats, these data prove that H-1PV can cross the blood-brain/tumor barrier from the blood into the tumor. In contrast to local therapy, no NS1 could be detected in any patient after intravenous ParvOryx injection (Table 3).

**Table 3:** Local and Systemic Responses to H-1PV Administration

Patient No.		Tissue Analyzed						
		Tumor^a^					Peripheral Blood^b^	
		Viral Parameters			Host Parameters		Specific Anti-H-1PV T Cell Responses	
		DNA	RNA	NS1	CTSB	CD45	Anti-NS	Anti-VP
G1-L1	1-01	+++	−	−	+	++	−	−
	1-02	+	++	+	+++	+++	NA	NA
	1-03	−	−	−	+	+	−	++
G1-L2	2-04	++	++	+	+	+	+++	+
	2-05	+	++	+	NA	NA	NA	NA
	2-06	+	+++	+	+++	++	NA	NA
G1-L3	3-07	+(++)	+(++)	+	++	+	NA	NA
	3-08	+(+)	+(++)	+(+)	+++	+++	++	+++
	3-09	+(+)	+(++)	+(++)	+++	+++	−	+
G3-L4	6-16	+++	+++	++	+++	+++	−	+++
	6-17	+(+)	++(+)	++(+)	+++	+	NA	NA
	6-18	+(+)	+(++)	+	++(+)	++(+)	NA	NA
G2-L2	4-10	−	+(+)	−	+++	++	+	++
	4-11	−	−	−	+	+++	−	−
	4-12	+	+	−	NA	NA	+	+++
G2-L3	5-13	+	+(+)	−	+	+	+	+++
	5-14	−	+(+)	−	++	++	+	+++
	5-15	+	−	−	+	++	−	−

NA, not analyzed.

a Presence of H-1PV nucleic acids and NS1 protein, cathepsin B (CTSB) expression, and lymphocytic infiltration were analyzed by FISH and immunofluorescence (IF) in several areas of the same tumor. Parentheses indicate variations, if any, in signal intensities (or number of positive cells) among different areas.

b Specific anti-H-1PV T cell responses were analyzed by using isolated patients’ peripheral blood mononuclear cells and viral peptide epitopes or full viral proteins as stimulants. For a detailed description of scoring criteria, see also Materials and Methods.

### Cathepsin B Induction after Local ParvOryx Administration

To further analyze H-1PV interactions with glioblastoma cells and their microenvironment, we examined in resected tissues the expression of cathepsin B (CTSB). In agreement with our results from a rat glioma model, where H-1PV infection led to CTSB overexpression,^29^ all G1-L3 and G3-L4 patients showed CTSB induction (Table 3; Figure 4E, left), in contrast to historical negative controls (Figure 4E, right). CTSB-overexpressing cells were observed mainly in tumor areas with high NS1 reactivity (Figure S2C). The majority of CTSB-overexpressing cells were identified as microglia/macrophages (Figure 4D, right). However, CTSB-positive non-macrophage cells were also detected (Figure 4D, right panel, arrow) and found to overexpress EGFR (data not shown), which hints at their tumor origin. For one G1-L1 patient (1-02), material was available from the resection of the primary tumor that had not been exposed to ParvOryx. Analyses of this control sample failed to reveal induction of CTSB and of the microglia/macrophage phagocytic competence marker Iba1, which was typically detected following virus application (Figures S3A and S3B). In intravenously injected patients, CTSB expression was lower than in intratumorally treated ones, albeit higher than in the historical controls screened (n = 10).

### Infiltration of Tumors with Activated Immune Cells

Prominent immune cell infiltrates were present in ParvOryx-treated patients (Table 3; Figure 4F, upper left) but were observed neither in historical negative controls (Figure 4F, upper right) nor in the primary tumor material (Figure S3C). Tumor-infiltrating leukocytes (TILs) expressed CD45 (Figure 4F, middle left) and the T lymphocyte-specific CD3 marker (Figure 4F, middle right). B lymphocytes were not detected, and NK cells were scarce. Staining for the mutually exclusive CD4 and CD8 co-receptors demonstrated that CD8 (Figure 4F, lower left) and to a lesser extent CD4 (Figure 4F, lower right)-positive T lymphocytes were the two major subpopulations. Tumor-infiltrating T cell activation status (Figures 4G–4I) was assessed by granzyme B (Figure 4G, left) and perforin (Figure 4G, right) staining. Both markers, indicative of T cell cytotoxic potential, were detected along with the immunostimulatory cytokines interferon-γ (IFN-γ) (Figure 4H, left) and interleukin (IL)-2 (Figure 4H, right). Accordingly, expression of CD25 (the alpha chain of the IL-2 receptor) was also demonstrated (Figure 4I, left). Expression of the co-stimulatory molecule CD154 (CD40L), a tumor necrosis factor protein superfamily member with a major role in antigen-presenting cell recognition, was also seen (Figure 4I, right). In situ FOXP3 analyses revealed only a few regulatory T (Treg) cells, scattered as single cells throughout the tumor, but not concentrated within the main immune infiltrate (Figure S4).

### Specific T Cell Responses in the Peripheral Blood of ParvOryx-Treated Patients

**Figure 5:**
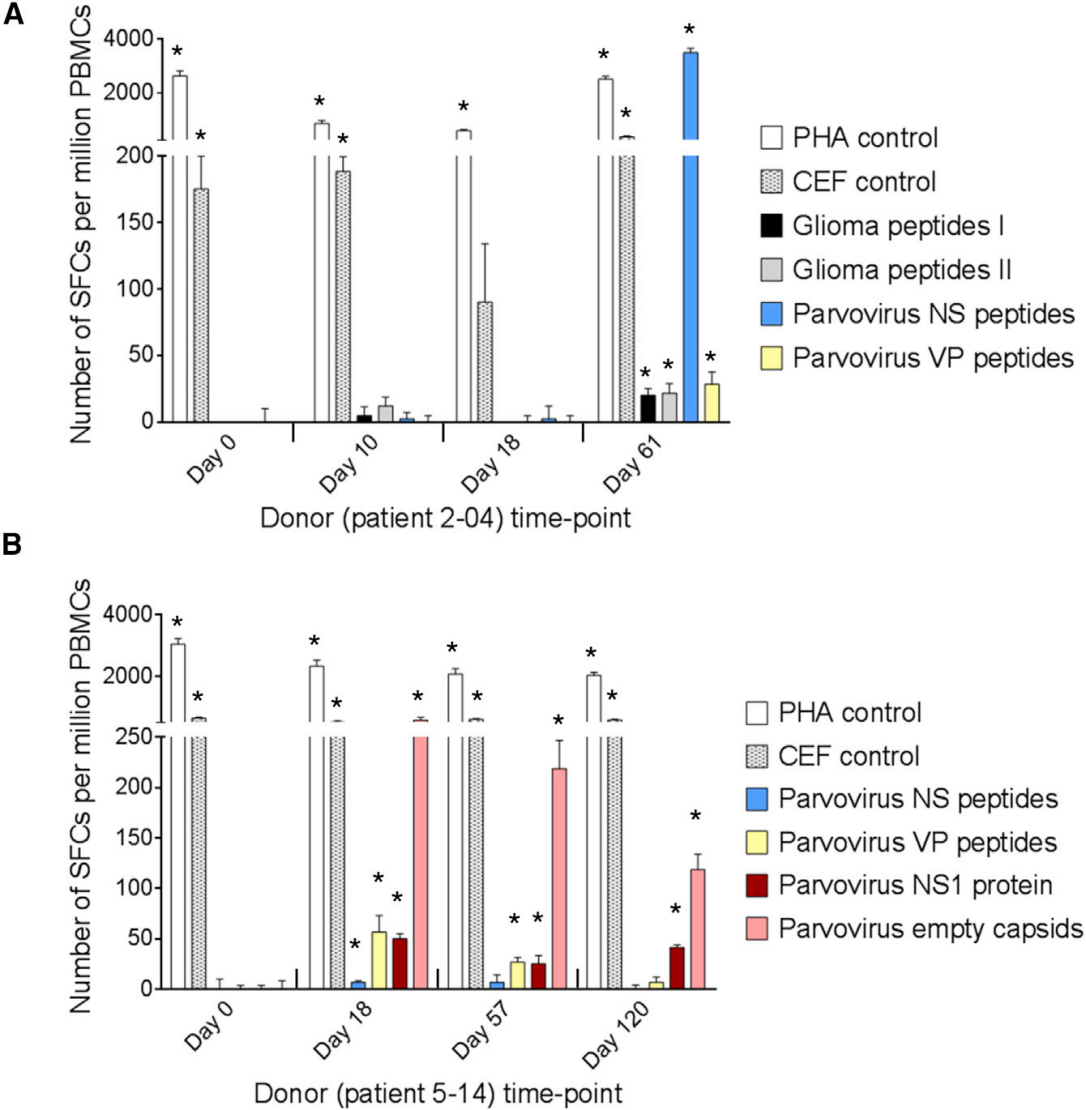
Evaluation of T Cell Responses to H-1PV and Glioma Antigens by IFN-γ ELISpot Assay (A and B) Cellular immune responses are shown for two patients treated with ParvOryx via (A) the intratumoral and intracerebral route (patient 2-04) or (B) the intravenous and intracerebral route (patient 5-14). PBMCs were isolated at the indicated days prior to (day 0) or after (days 10–120) treatment. After incubation with appropriate stimulants, IFN-γ-producing spot-forming cells (SFCs) were counted. The test stimulants were viral or glioma peptides (Table S3) or full-length viral proteins (NS1 or empty capsids made of VP1 and VP2). Phytohemagglutinin (PHA) and cytomegalovirus, Epstein-Barr virus, and influenza virus (CEF) peptide pools served as positive control stimulants. Negative control values (unstimulated cells) ranged from 0 to 21 SFCs per million PBMCs and were subtracted from the corresponding stimulated sample values. Means (columns) and SEMs (bars) of triplicate measurements are shown. Asterisks denote statistical significance (*p ≤ 0.05; mean SFC − 2 SEMs > 2× negative control).

Twelve patients were tested for induction of virus-specific cellular immune responses by measuring the reactivity of their T cells to viral antigenic determinants in IFN-γ ELISpot assays. The stimulants used were full-length viral proteins (NS1, empty capsids made of VP1/2) and/or peptide derivatives previously shown on a panel of glioma cell lines to be presented by HLA-I (Table S3). Nine of the 12 tested patients were found to mount a significant antiviral T cell response against NS (6 patients) and/or VP (all 9 patients) epitopes (Table 3). Virus-reactive T cells were detected in patients of all treatment groups at all dose levels within 2–8 weeks of the first ParvOryx treatment and persisted for several months (Figure 5). Interestingly, the four patients in whom viral transcripts were not detected by FISH (patients 1-01, 1-03, 4-11, and 5-15) also failed to develop an NS1-specific T cell response (Table 3). This argues for the dependence of this response on de novo NS1 production by infected tumor cells, because the genome-linked pre-existing copy of the NS1 polypeptide present on the outside surface of the input virion was removed during ParvOryx purification through DNase digestion of the externally located tether sequence to which NS1 is covalently attached. To further determine activated lymphocyte specificity, we tested shorter (9-mer) and single viral peptides. This led to identifying distinct virus-specific cytotoxic T lymphocyte (CTL) epitopes. Because the HLA I-presented peptides detected in H-1PV-infected human glioma cell lines include a number of putative glioma antigen epitopes (Table S3), we tested for T cell reactivity against these glioma peptides in patients whose HLA type closely matched that of glioma cell lines. As exemplified in Figure 5A, three out of six such patients showed a low but significant T cell response to glioma antigens.

## Discussion

ParvOryx01 was a first-in-human trial for the use of H-1PV in recurrent glioblastoma patients. Despite disparities within the trial population that were expected from the rather wide inclusion criteria regarding gender, age, tumor size, and previous treatments, ParvOryx was generally well tolerated over the entire range of investigated doses. Thus, the primary objective of safety and tolerability was met. There were no signs of systemic inflammation, excessive immune activation, or main organ toxicity. The absence of detectable mutations in the genomes of viruses recovered from resected tumors argued against the emergence of adapted variants of H-1PV during the time interval studied, in line with their similar Vg/PFU ratios compared to the input virus. The SUSAR observed in one G3-L4 patient remained a singular event, and although the first symptoms appeared shortly after the second ParvOryx administration, no unequivocal causal connection could be established between the pathological CSF, the radiological diagnosis of hydrocephalus, and the study drug. In particular, intraventricular H-1PV propagation and virus-related pathology such as active encephalitis and/or meningitis could be ruled out, and most likely the event was due to an aberrant immune response, potentially triggered by the patient’s individual immune propensity in the CNS. Therefore, it was not ruled as DLT, and the DSMB as well as the regulatory bodies allowed the trial to continue. As no further events occurred, MTD of ParvOryx was not reached.

ParvOryx01 demonstrated for the first time in humans the ability of H-1PV to pass, in a dose-dependent manner, from a brain tumor to the bloodstream and vice versa. This confirms preclinical findings in rats, showing systemic availability of the virus after intracerebral injection.^22^, ^27^ Given that patients with brain tumors have a leakier-than-normal vasculature, this observation opens new therapeutic opportunities, notably for glioblastoma, which is characterized by early intracerebral tumor cell migration requiring additional systemic delivery of therapeutics.

Our analysis of the H-1PV distribution after local injection demonstrates that a single, slow injection through a standard catheter resulted in excellent targeting of the inoculum to the tumor area and wide distribution of H-1PV through the tumor tissue. Future clinical trials using ParvOryx for this or other tumor types might thus avoid exploring more complicated methods for local administration such as convection-enhanced delivery.

The presence of viral RNA in tumor cells after intravenous ParvOryx infusion indicates that systemic therapy is an option, and should take into account that NS1 production was dose dependent and detected only after local injection at L2 or above. Observations favorable to systemic therapy include: (1) good predictability of the drug’s pharmacokinetics, minimizing the risk of unintended overdosing and exposure-related side effects; (2) a high volume of distribution of H-1PV after intravenous injection, suggesting broad dissemination to various tissues,^26^ and hence the potential applicability to various malignancies (although the uncontrolled loss of H-1PV to non-target organs could be an obstacle to efficient treatment); and (3) absence of biohazard risks for the general population after administration of ParvOryx within the investigated dose range. In future trials, precautions for ParvOryx-treated patients can thus be considerably reduced.

H-1PV compares favorably with other tested OVs in that pre-existing H-1PV-specific antibodies are absent in the general population. Within 10 days of ParvOryx administration at the highest doses tested, H-1PV-specific antibodies appeared, providing at least a 10-day window for uninhibited booster reapplication. However, future prolonged ParvOryx treatment schedules will have to take into account the timing of the appearance of neutralizing antibodies, accelerating H-1PV clearance from the blood.

Estimates of clinical efficacy, a secondary endpoint, must be considered with caution because ParvOryx01 included only 18 rather heterogeneous patients and was conducted as a dose escalation study with different routes of administration. In our patient cohort, clinical response did not depend on the dose or mode of ParvOryx administration. Objectively, a PFS of 15.9 weeks and an OS of 464 days compared favorably with published data of meta-analyses of recurrent glioblastoma patients and were in the range of recently reported positive results from a trial using a replication-competent armed retrovirus.^14^ A possible confounder is the effect of repeated surgery, which in several small, single-center studies and one recent multicenter analysis^30^ seemed to improve outcome. In contrast, in a larger comprehensive analysis reported by the North American Brain Tumor Consortium in 2007, this effect was minimal: the median PFS ranged from 7.9 (no surgery) to 8.3 weeks (with surgery), and OS at 6 months was 51% without or 56% with tumor removal.^31^

During study planning, a decision was made against taking biopsies to confirm the diagnosis prior to virus injection, based on a risk-benefit analysis. Histology from resected tumor confirmed recurrent glioblastoma in all cases, so this approach proved correct. However, taking tumor samples before ParvOryx injection would have facilitated the comparison and interpretation of in situ histological and immunological findings and can be considered in future studies. Nonetheless, it was possible to obtain primary tumor material from one of the patients. While keeping in mind that the ParvOryx-treated recurrent tumor was subjected to radiotherapy and chemotherapy prior to virus exposure, this primary tumor material served as a pretreatment “no virus” control, together with the panel of historical recurrent glioblastoma cases screened. Regarding the timing of surgery, tumor removal after treatment provided highly informative material, but a 9-day incubation period before resection was probably too short for the virus to express its full antineoplastic potential.

Histopathological examination of resected tumors revealed the presence of multiple necrotic areas, a hallmark of glioblastoma. Clusters of infected NS1-expressing tumor cells were found near such areas, in so-called palisades of active tumor tissue, but whether the virus actually contributes to necrosis induction requires further investigation. Tumors from ParvOryx-treated patients that were NS1- and/or viral RNA-positive displayed markers of local activation of tumor-associated microglia/macrophages, such as CTSB.^32^ Activated microglia can in turn secrete factors that efficiently kill glioma cells in culture, under conditions where both neurons and normal astrocytes show unimpaired viability.^33^ Furthermore, CTSB production by activated microglia is associated with glioma, but not normal cell, apoptosis.^34^ In human glioma cells, H-1PV infection leads to CTSB dysregulation inducing cell death.^29^ Accordingly, a minor fraction of CTSB-overexpressing cells in ParvOryx-treated patients displayed a non-macrophage EGFR-positive phenotype, suggesting that endogenous CTSB induction may also contribute to tumor cell killing.

Tumors from six ParvOryx-treated patients displayed strong leukocytic infiltration, clearly different from negative untreated controls, and in all but one tumor the presence of dense leukocytic infiltrates coincided with the detection of H-1PV DNA, RNA, and NS1 protein. The predominant leukocytic cell populations were CD8^+^ and CD4^+^ T lymphocytes. TILs are reported to occur mainly in glioblastomas of the mesenchymal transcriptional class, whereas significant TIL depletion has been observed in classical glioblastoma cases.^35^ Interestingly, none of the heavily infiltrated H-1PV-infected tumors could be assigned to the typical mesenchymal glioblastoma subgroup, further arguing for a role of ParvOryx treatment in inducing intratumoral TIL accumulation. Because a prominent population of immunoinhibitory Treg cells is typically present in the glioblastoma microenvironment, the action of tumor-infiltrating effector immune cells is often suppressed.^36^ In contrast, the CD8^+^ T cell-positive tumors of ParvOryx01 patients had very few tumor-invading Treg cells, in line with recent observations that H-1PV can inhibit the suppressive activity of Treg cells in vitro.^37^ Further support for a contribution of ParvOryx treatment to the establishment of an immunogenic intratumoral milieu comes from the detection in locally treated tumors of several markers of immune cell activation, namely perforin, granzyme B, IFN-γ, IL-2, CD25, and CD40L.

The results of IFN-γ ELISpot assays in PBMCs revealed an early induction of persistent CTL responses to structural and/or non-structural viral antigens. Even though virus-triggered immune responses might interfere with virus replication, it has been reported that cellular immune reactions induced by other OVs correlate with responsiveness to treatment by promoting antitumor immunity.^38^ Therefore, the observed H-1PV-specific T cell responses appear in a clinically favorable light, especially because a small but significant population of CTLs also recognized peptide epitopes derived from known glioma antigens. Although specificity for patients’ gliomas could not be tested, the appearance of these CTLs supports an H-1PV-elicited antitumor cellular immunity.

In conclusion, the ParvOryx01 trial data confirm H-1PV safety and tolerability. They provide evidence of a lack of ectotoxicity, H-1PV ability to cross the blood-brain/tumor barrier, and favorable (progression-free) survival compared with historical controls. Finally, this trial points to H-1PV capacity for establishing an immunogenic tumor microenvironment, making H-1PV an interesting candidate for further clinical development.

## Materials and Methods

### Study Design

ParvOryx01 was an open, non-controlled, three-group, intra-group dose escalation, single-center study using a good manufacturing practice (GMP)-grade pharmaceutical formulation of H-1PV (ParvOryx). Its design is reported in Geletneky et al.^24^ and depicted in Figure 1. Primary objectives included ParvOryx safety and tolerability assessment, MTD determination, and viremia and H-1PV shedding investigation. Secondary objectives were proof-of-concept, PFS6, and OS6. Whenever applicable, patients were followed up for OS beyond the regular study follow-up period of 6 months by means of telephone interviews or continuing visits of the trial center.

ParvOryx01 was registered in clinical trials databases (EudraCT: 2011-000572-33 and ClinicalTrials.gov: NCT01301430), conducted according to the principles of the Declaration of Helsinki, and approved by the German competent authority PEI and the Ethics Committee of the Medical Faculty Heidelberg. A DSMB regularly reviewed treated patient safety data and gave recommendations on trial progression.

Initially, it was planned to treat an equal number of patients in each treatment arm. This plan was revised, considering that patients having received ParvOryx intratumorally at the third dose level (G1-L3, 1E9 PFU) showed levels of systemic exposure to H-1PV similar to those expected (on the basis of animal experiments) for the lowest dose subgroup of the intravenous arm. Therefore, after approval of a protocol amendment, the three patients originally scheduled for intravenous treatment at G2-L1 were assigned to G3-L4 and received instead ParvOryx intratumorally at a virus dose (5E9 PFU in total) five times higher than the G1-L3 patients (Table S4). The protocol required completion of the intratumoral treatment in G1 before continuing with treatment in G2 and G3. Hygiene measures included the obligation to remain strictly isolated in the study center until the first occurrence of H-1PV-specific antibodies or until shed Vg were no longer detected in feces, urine, or saliva. The medical staff and visitors observed additional, predefined hygiene measures. After patient discharge, four ambulatory follow-up visits were scheduled (day 28 and months 2, 4, and 6) (Tables S5 and S6).

The investigated safety and tolerability parameters were: (serious) AEs, 12-lead electrocardiograms, body temperature, blood pressure, heart rate, clinical chemistry, hematology, and clotting. Vg concentrations in blood, urine, saliva, and feces were determined by quantitative real-time PCR at screening, daily between study days 1 and 18, and at each follow-up visit. In G2, two additional blood samples were taken on each day of intravenous administration according to a preset schedule. LLOQs were 40 Vg/μL for blood, 20.9 Vg/mg for feces, 8.57 Vg/μL for urine, and 9E4 Vg/swab for saliva. Serum antibody titers were measured at screening, daily between study days 1 and 18, and at each follow-up visit. Serum antibody titers were determined with an HI assay. PFS was assessed by Macdonald criteria.^39^

### Fluorescence In Situ Hybridization

The FISH assay^40^, ^41^ used viral nucleic-acid-specific digoxin-tagged locked nucleic acid (LNA) hybridization probes custom-designed by Exiqon (Vedbaek, Denmark) (Table S7). The sense probe recognizes both virion genomes and the negative strand of viral DNA replicative forms. The antisense probe detects viral mRNA and the positive strand of DNA replicative forms. The signals generated by the antisense probe, being mostly RNase-sensitive, were used as indicators of viral transcript synthesis. For quantitative analysis of positive signals, the Fiji image processing package^42^ ImageJ^43^ was used. Custom macros were developed by Dr. D. Krunic (German Cancer Research Center, Heidelberg, Germany), and image analysis was done with constant processing settings. Results were presented as average intensities of positive signals (in arbitrary units [a.u.]) per microscope observation field (diameter of field of view [dFOV] = 1,000 μm). The background fluorescence of historical negative controls defined the cutoff between positive and negative signals. Signal intensity within the ranges 11,500–30,000 a.u., 30,000–50,000 a.u., and >50,000 a.u. was scored as +, ++, and +++, respectively.

### Quantitative Real-Time PCR Analysis of Tumors

Viral nucleic acids were extracted from paraffin-embedded tumor tissue (∼10 mg) with the AllPrep DNA/RNA FFPE Kit (QIAGEN, Hilden, Germany). For quality assurance, positive-matrix (spiked with defined viral DNA and RNA) and negative-matrix controls were used. Extracted nucleic acids were quantified with a NanoDrop 2000 (Thermo Scientific, Darmstadt, Germany). For DNA quantification, samples were mixed with TaKaRa Premix ExTaq Mastermix (TAKARA Bio, Kusatsu, Japan) containing ROX, sequence-specific primers, and NS-probe ensuring DNA (nt 1,079–1,219) amplification.^44^ For RNA quantification, samples were mixed with TaqMan RNA-to-CT 1-Step Kit Mastermix (Life Technologies, Darmstadt, Germany) containing reverse transcriptase, sequence-specific primers (reverse primer: 5′-GGCGTACTTCTCGGAGTCAGA-3′, forward primer: 5′-GAGCGCAGTGGATGACATGA-3′) and probe (5′-[6FAM]CAAAAAGTTCAATGCGCTCA[MGB]-3′) ensuring cDNA (nt 491–2,032 exon-exon region) amplification. Viral nucleic acid concentrations were expressed as viral DNAs/μg total DNA or viral RNAs/μg total RNA.

### Immunofluorescence

The mouse monoclonal antibody 3D9 specific for H-1PV NS1 protein was provided by Dr. N. Salomé (DKFZ [German Cancer Research Center], Heidelberg, Germany). Various commercially available primary antibodies were used to detect CTSB and immune cell (activation) markers (Table S8). NS1-positive cells per observation field (dFOV = 2,000 μm) were counted, and their abundance was scored as +, ++, or +++, if 1–10, 10–50, or ≥50 positive cells were detected, respectively. The CTSB-specific signal was scored as +, ++, or +++ when the number of CTSB-positive cells per microscope observation field (dFOV = 1,000 μm) was ≤5, 5–10, or ≥10, respectively. Only CTSB-overexpressing cells (mean signal intensity above the cutoff determined on historical negative controls) were taken into account. For quantitative analysis of CTSB signal intensity, the Fiji ImageJ software was used (see above), and automated analysis was performed with purpose-developed macros (Dr. D. Krunic, DKFZ) and constant processing settings. Lymphocytic tumor infiltration was scored as +, ++, or +++ if the number of TILs per microscope observation field (dFOV = 1,000 μm) was ≤10, 10–20, or ≥20, respectively.

### Immunohistochemistry

Routine protocols and an automated random-access staining platform (Ventana Medical Systems, Basel, Switzerland) were used for CD45, CD3, CD4, CD8, CD68, and FOXP3 detection.

### IFN-γ ELISpot Assay

PBMCs were prepared from whole blood collected from patients before and at intervals after pre- and post-resection ParvOryx administration. Responding T cells were quantified as spot-forming cells per million PBMCs, using the IFN-γ enzyme-linked immunospot (ELISpot) assay (ProImmune, Oxford, UK). Test stimulants included purified H-1PV NS1 protein,^45^ empty capsids made of VP1/2^46^ (10 μg/mL), and pools of synthetic peptides (5 μM) from H-1PV NS or VP proteins or known glioma antigens (Table S3). Test peptides were identified beforehand with the ProPresent Antigen Presentation Assay (ProImmune) as being HLA I-presented on a panel of H-1PV-infected glioma cell lines (U138, U87, A172, U373, and NCH89).

### Detecting Infectious H-1PV

Infectious parvovirus titers in blood and tumor were determined by plaque assay. Samples (200 μL of blood or 25 mg of tumor brought to 500 μL total volume with MEM medium without fetal bovine serum [FBS]) were homogenized by three freeze-thaw cycles and sonicated at 48 W for 1 min in a Sonorex Super 10 P ultrasonic homogenizer (Bandelin, Germany). Aliquots (500 μl) of serial dilutions were inoculated onto 60% confluent monolayers of newborn kidney cells (NB-324K). Cultures were processed essentially as described by Tattersall and Bratton,^47^ and virus titers were expressed in PFU per milliliter of blood or milligram of tumor. The LLOQ was 5 PFU/mL blood or 40 PFU/g tumor. When the titer was below 3 PFU/200 μL blood or 3 PFU/25 mg tumor, the original sample was propagated on RG-2 rat glioma cells (three cycles of 5-day incubation) prior to plaque titration. The Vg/PFU ratio of propagated tumor- and-blood-derived viruses was determined after removal of non-packaged DNA by DNase treatment and found to be similar to that of the input virus (around 1E3).

### H-1PV DNA Sequencing

After in vitro multiplication, viruses recovered from resected tumor homogenates were subjected to DNA extraction using the QIAamp MinElute Virus Spin Kit (QIAGEN, Hamburg, Germany). Two-fold coverage sequencing of the full genome (except for the terminal hairpins) was performed under ISO 17025 conditions by Eurofins Medigenomix (Ebersberg, Germany), using the chain termination method.^48^

### Vg Quantification in Body Fluids

DNA extracted with commercial kits (QIAGEN, Hilden, Germany or Epicenter, Madison, WI, USA) from blood, saliva, urine, and feces was analyzed by quantitative real-time PCR. The assay was validated and performed by BSL Bioservice (Munich, Germany) in a LightCycler 480 (Roche, Mannheim, Germany). For calibration, two negative controls and seven duplicate Vg spikes were used (5E1–5E7 genomes per PCR). The primers and fluorogenic Vg detection probe were: forward primer (5′-GCG CGG CAG AAT TCA AAC T-3′), reverse primer (5′-CCA CCT GGT TGA GCC ATC AT-3′), probe (5′-[6FAM]ATG CA*G CCA* GA*C A*GT TA[TAMRA]-3′ [*, functionalized LNA]). The reaction was initialized for 10 min at 95°C, followed by 45 cycles of 95°C for 15 s and 60°C for 60 s, and final extension for 10 min at 40°C. Vg concentrations were expressed per microliter (blood, urine), swab (saliva), or milligram (feces).

### HI Assay

Antibody titers were measured by inhibition of virus-mediated hemagglutination. The HI assay was validated and performed by Labor Enders (Stuttgart, Germany), using 1:2 serial dilutions of patient serum depleted of nonspecific hemagglutination factors by prior incubation with 5% (v/v) chicken erythrocytes. Test samples were supplemented with H-1PV (approximately 5E9 capsids/well) and chicken erythrocytes (0.125%). Antibody titers were determined as the highest serum dilution causing complete HI.

### Neutralizing Antibody Assay

Virus-specific neutralizing antibodies were detected by measuring the ability of serum to inhibit lytic infection of permissive NB-324K cells by H-1PV. Cell viability was determined by crystal violet staining and photometric quantification of residual cells 4 days post-infection (0.1 PFU/cell) in the presence and absence of serially diluted patient serum. Dose-response curves were calculated with SigmaPlot and the 4PL model. The neutralizing antibody titer was defined as the serum dilution causing 50% inhibition of virus-induced toxicity.

### Statistical Planning

The trial was planned as a dose-finding trial with a three-at-once scheme with escalation to the next dose when no dose-limiting event occurred and transitioning to continual reassessment^49^ after the first dose-limiting event.

### Statistical Analysis

Continuous variables were tabulated per subgroup as means with SD; counts were tabulated as absolute frequencies per subgroup. Time-to-event data were estimated per subgroup and per group using the Kaplan-Meier method. Shift-tables were generated for all laboratory values per subgroup and visit. Viral genome concentration was scatterplotted on a logarithmic scale against time for G2. All analyses were pre-specified in a statistical analysis plan, which was finalized before the study database was closed.

## Author Contributions

J. Hajda, K.G., J.R., J. Hüsing, K.J., O.K., M.D., and B.H. designed this study. K.G., A.U., J.-O.N., and T.S. were responsible for the conduct of the trial. K.G., A.U., J. Hajda, B.B., and A.J.B. acquired and/or interpreted clinical data. D.C., A.v.D., V.D., B.L., I.K., A.L.A., M.R., A.J., V.F., S.L., J.F., W.B., R.B., and E.T.-L. performed laboratory measurements. B.L., A.L.A., J.R., I.K., D.C., K.G., J. Hajda, J.F., R.B., O.K., and M.D. analyzed and interpreted the laboratory results. J. Hüsing was responsible for statistical analysis. K.G., J. Hajda, A.L.A., B.L., J. Hüsing, J.R., and A.U. wrote the manuscript and/or critically reviewed its intellectual content. All authors approved the final version of the manuscript.

## Conflicts of Interest

The ParvOryx01 clinical trial was sponsored by ORYX GmbH & Co. KG. The sponsors had no role in the decision to publish or in the writing of the manuscript. O.K., M.D., and B.H. are employees of ORYX GmbH & Co. KG. K.G., A.L.A., B.L., I.K., and J.R. are co-inventors in various patents/patent applications relating to the content of this work.

## Supplemental Information

Document S1. Figures S1–S4 and Tables S1–S8

Document S2. Article plus Supplemental Information
