# Supplementary material for: Oncolytic H-1 Parvovirus Shows Safety and Signs of Immunogenic Activity in a First Phase I/IIa Glioblastoma Trial
Source: Mol Ther. 2017 Aug 24;25(12):2620–34. doi: 10.1016/j.ymthe.2017.08.016 (PMC5768665; doi:10.1016/j.ymthe.2017.08.016)
Supplement: Document S1. Figures S1–S4 and Tables S1–S8 [file mmc1.pdf]

YMTHE, Volume *25*

Supplemental Information

Oncolytic H-1 Parvovirus Shows Safety and Signs of Immunogenic Activity in a First Phase I/IIa Glioblastoma Trial

Karsten Geletneky, Jacek Hajda, Assia L. Angelova, Barbara Leuchs, David Capper, Andreas J. Bartsch, Jan-Oliver Neumann, Tilman Schöning, Johannes Hüsing, Birgit Beelte, Irina Kiprianova, Mandy Roscher, Rauf Bhat, Andreas von Deimling, Wolfgang Brück, Alexandra Just, Veronika Frehtman, Stephanie Löbhard, Elena Terletskaia-Ladwig, Jeremy Fry, Karin Jochims, Volker Daniel, Ottheinz Krebs, Michael Dahm, Bernard Huber, Andreas Unterberg, and Jean Rommelaere

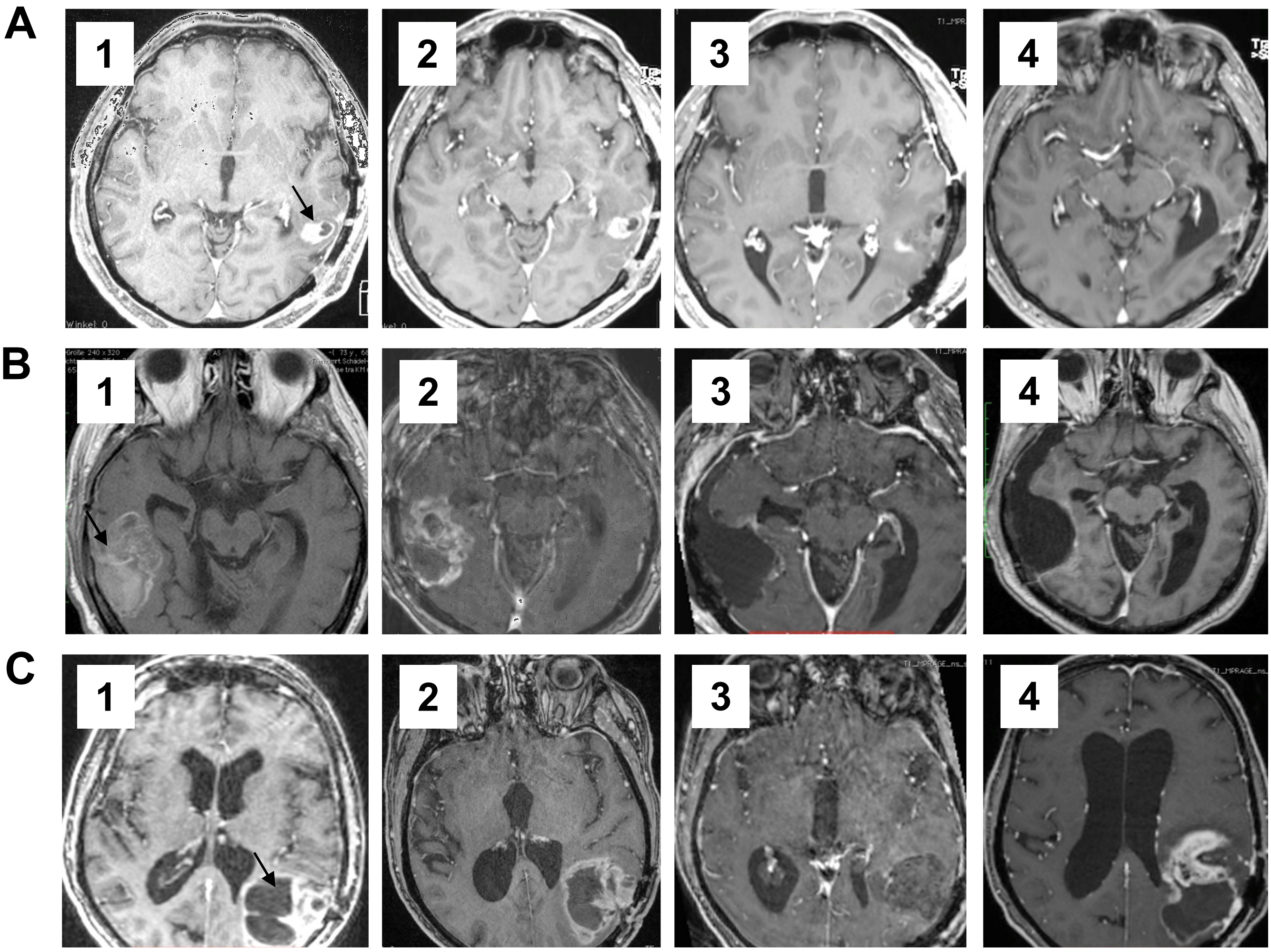

Figure S1. Examples of regular study MRIs of patients from different treatment groups

Upper panel (A1 to A4): patient 1-03, left temporal tumor (arrow). MRIs before treatment (A1), on day 9 after intratumoral virus injection prior to surgery (A2), on day 11 one day after surgery (A3) and after 6 months (A4) demonstrating stable disease. Middle panel (B1 to B4): patient 4-10, right temporal tumor (arrow). MRI before treatment (B1), on day 9 after the first intravenous virus injection prior to surgery (B2), on day 11 one day after surgery (B3) and after 2 months (B4) demonstrating that the patient died of unrelated causes (pneumonia). Lower panel (C1 to C4): patient 6-17, left temporo-occipital tumor (arrow). MRI before treatment (C1), on day 9 after intratumoral virus injection prior to surgery (C2), on day 11 one day after surgery (C3) and after 2 months (C4) demonstrating massive tumor progression.

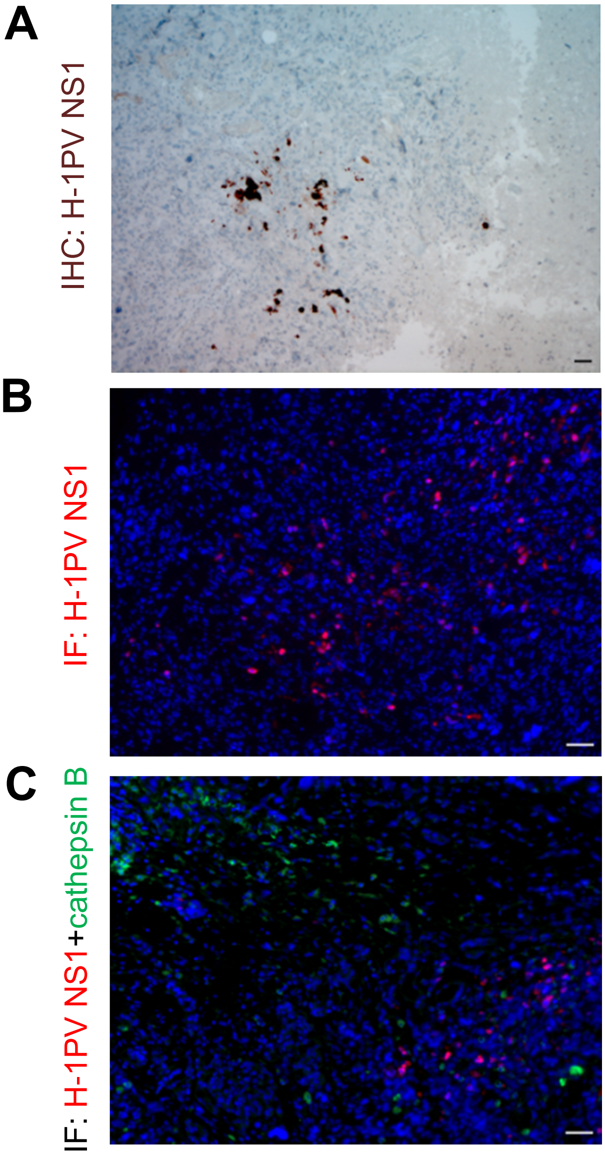

Figure S2. Clustering of NS1-positive cells within solid tumor areas after local ParvOryx administration
Tumor (patient 6-17) was resected 9 days after intratumoral ParvOryx (2.5E9 PFU) administration, and paraffin-embedded tumor tissue was subjected to analysis. (A) Immunohistochemical and (B) immunofluorescent NS1 protein detection using the 3D9 NS1-specific antibody revealed clusters of cells with high NS1 reactivity. (C) Strong cathepsin B expression (green) was seen in NS1-positive (red) tumor regions. Scale bars, 50 µm.

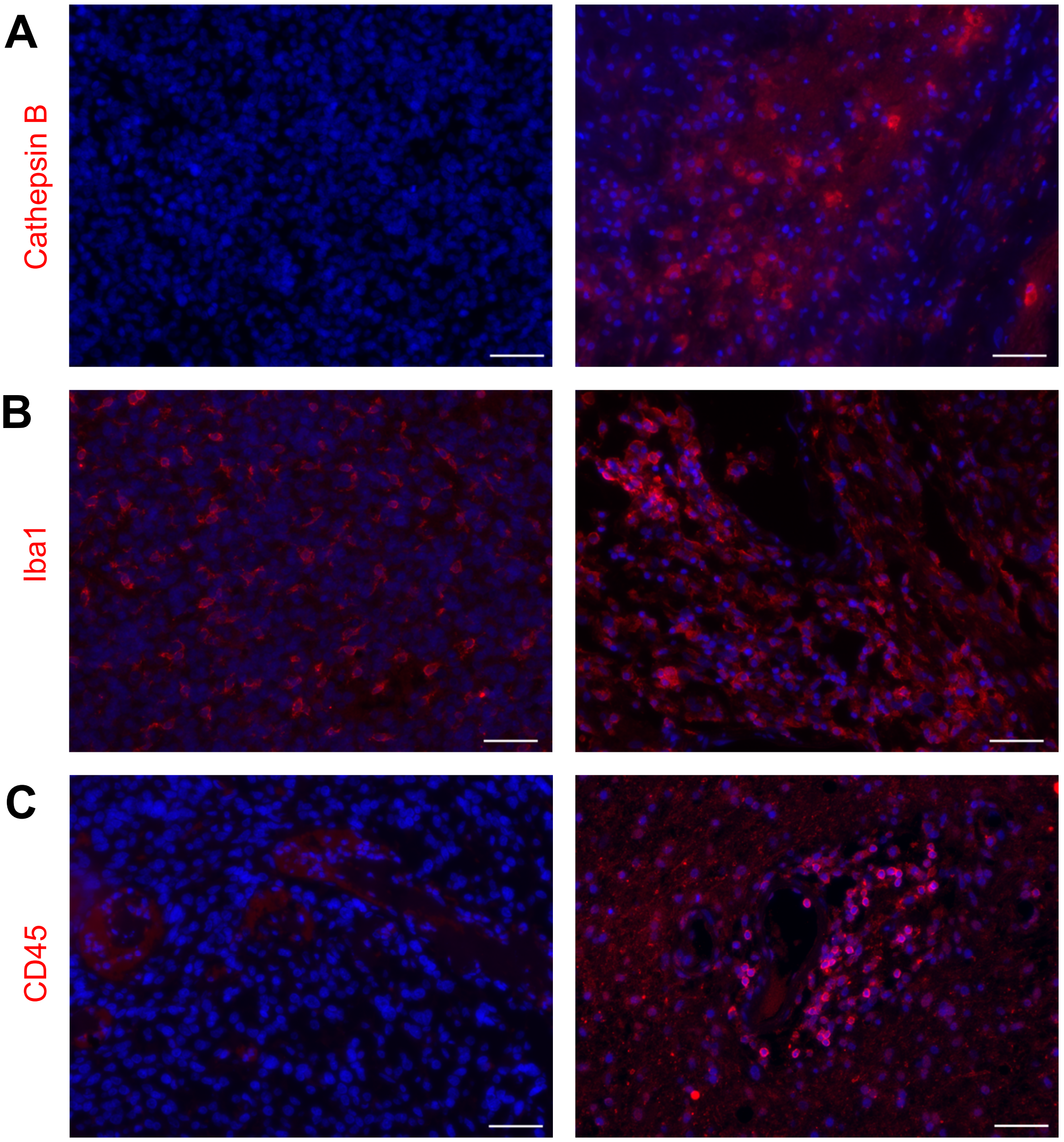

Figure S3. Microglia/macrophage activation and leukocytic infiltration in a ParvOryx-treated recurrent glioblastoma compared with the corresponding primary tumor

Recurrent glioblastoma (patient 1-02) was resected 9 days after intratumoral ParvOryx (5E5 PFU) administration, and paraffin-embedded tumor tissue was subjected to immunostaining (right panels). For comparison, the same analysis was performed with control samples from the resected primary tumor of the same patient (left panels). This primary tumor – which was not exposed to ParvOryx – gave rise, after resection to the recurrent tumor which was treated with ParvOryx in the framework of the present trial. (A) In contrast to the primary tumor, the ParvOryx-treated recurrent glioblastoma showed significant cathepsin B induction. (B) In comparison to pretreatment primary material, an increased microglia/macrophages phagocytic competence was noted after virus application. (C) Similarly, perivascular leukocytic infiltrates were detected only in the post-ParvOryx-treatment tumor tissue. Scale bars, 50 µm.

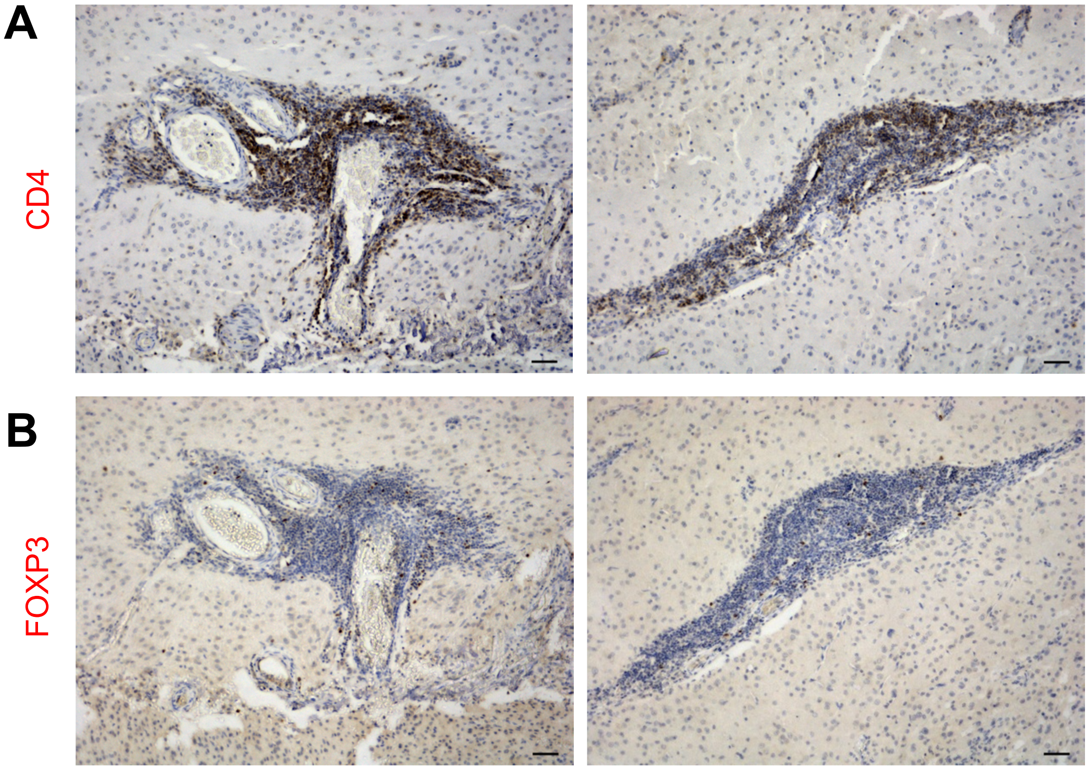

Figure S4. Scarce Treg cell detection within tumor immune cell infiltrates

Recurrent glioblastoma (patient 3-08) was resected 9 days after intratumoral ParvOryx (5E8 PFU) administration, and tumor-infiltrating immune cell populations were analyzed in situ through immunohistochemical staining. Prominent $CD3^+$ infiltrates were detected within the tumor and found to consist of both $CD8^+$ (not shown) and (A) $CD4^+$ T cells. (B) $FOXP3^+$ Treg cells represented only a very minor subpopulation within the $CD4^+$ tumor immune cell infiltrate. Scale bars, 50 µm.

Table S1. Main characteristics of serious adverse events (SAEs)

Cohort	Subject ID	Preferred term	Start day	End day	Severity	Outcome	Relationship to ParvOryx
G1-L1	1-02	Cerebrospinal fistula	101	117	Severe	Resolved	Unlikely
		Post-operative wound infection	101	117	Severe	Resolved	Unlikely
G1-L2	2-04	Deep vein thrombosis	95	189	Severe	Resolved	Unlikely
		Pneumonia	95	189	Severe	Resolved	Unlikely
		Pulmonary embolism	95	189	Severe	Resolved	Unlikely
	2-06	Cystitis	80	87	Severe	Resolved	Unlikely
		Urosepsis	116	Unknown	Severe	Unknown	Unlikely
G1-L3	3-07	Wound dehiscence	31	129	Severe	Resolved	Unlikely
		Upper-limb fracture	160	198	Severe	Resolved	Unlikely
G2-L2	4-10	Fall	28	28	Severe	Resolved	Not related
		Head injury	28	28	Severe	Resolved	Not related
		Subdural hygroma	56	Unknown	Severe	Unknown	Unlikely
		Reduced level of consciousness	71	98	Severe	Not resolved	Unlikely
		Pneumonia	Unknown	98	Fatal	Fatal	Unlikely
G3-L4	6-16	Reduced level of consciousness	12	185	Severe	Not resolved	Possible
		Secondary complications initiated by hydrocephalus	15	185	Fatal	Fatal	Possible
		Occlusion of ventricular catheters due to high protein levels in CSF	16	Unknown	Life-threatening	Unknown	Possible
	6-18	Reduced level of consciousness	11	102	Severe	Not resolved	Unlikely
		Convulsion	16	Unknown	Severe	Unknown	Unlikely

Table S2. Frequency of adverse events sorted by treatment cohort

AE subset	G1-L1 Frequency Count		G1-L2 Frequency Count		G1-L3 Frequency Count		G2-L2 Frequency Count		G2-L3 Frequency Count		G3-L4 Frequency Count	
	P	E	P	E	P	E	P	E	P	E	P	E
Fatal events	.	.	1	1	.	.	1	1	.	.	2	2
Serious events	1	2	2	5	1	2	1	5	.	.	2	5
Related events	.	.	.	.	.	.	1	1	.	.	1	4
All events	3	19	3	32	3	25	3	28	3	23	3	58

P, no. of patients; E, no. of events.

Table S3. HLA class I-presented viral and glioma peptides on H-1PV-infected human glioma cells[a]

Viral protein	Peptide sequence/position (aa)[b]
NS1; NS2	**NENVQLNGKDIGW/32-44**
NS1	**NENITVVRI/486-494**
VP1	AHIFVNQA/118-125
VP1; VP2	**SDGTETNQPDTGIANARVERSAD/145-166; 2-24**
	**TEQQGAGQDAIKVY/301-314; 159-172**
**Glioma protein**	
MAGEA1	**KEADPTGHSY/160-169**
CSPG4	**TMLARLASA/21-29**
	DTPLGTVALLQ/763-773
SART1	KLGLKPLEV/129-137
	KTSSGDASSLSIE/109-121
SART3	TVKDLRLVTNR/790-800
EPHA2	NIMNDMPIY/57-65
PTPRZ1	VTGKVFAGIPTV/1344-1355
MICA/B	**HLDGQPFLRY/50-59**
NRCAM	KVQALNDM/801-808

[a] Cells from a panel of 5 glioma lines showing broad HLA-class I allele expression were infected with H-1PV and processed for the identification of HLA I-presented peptides by immunoaffinity separation and sequencing mass spectrometry (see also Methods).

[b] Presented peptides identified as homologous (Expect Value <0.3) to fragment sequences (amino acid [aa] position) from H-1PV proteins[1] or known glioma antigens.[2] Peptides with an Expect Value <0.05, which is indicative of identity, are marked in **bold**. Peptide pools and individual peptides were used as stimulants in IFN-γ ELISpot assays. PBMCs were seeded on multi-well PVDF membrane plates pre-coated with anti-IFN-γ capture antibodies and incubated with (or without) a test or control stimulant for 18 h. Phytohemagglutinin (PHA, 25 µg/ml) and the CEF pool of common $CD8^+$ T cell epitopes (2.5 µM) were used as positive control stimulants.

1. Rhode, S.L. III, Paradiso, P.R. (1983). Parvovirus genome: Nucleotide sequence of H-1 and mapping of its genes by hybrid-arrested translation. J Virol. *45*, 173-184.

2. Dutoit, V., Herold-Mende, C., Hilf, N., Schoor, O., Beckhove, P., Bucher, J., et al. (2012). Exploiting the glioblastoma peptidome to discover novel tumour-associated antigens for immunotherapy. Brain. *135*, 1042-1054.

Table S4. Main characteristics of dosing schedule

GROUP 1

Escalation level	Study day	Dose	Route of administration	Duration
**Level 1** Total dose: 1E6 PFU	Day 1	5E5 PFU	intratumoral (via catheter)	15 min
	Day 10	5E5 PFU	intracerebral (direct injection at several locations in resection wall)	15–30 min
**Level 2** Total dose: 5E7 PFU	Day 1	2.5E7 PFU	intratumoral (via catheter)	15 min
	Day 10	2.5E7 PFU	intracerebral (direct injection at several locations in resection wall)	15–30 min
**Level 3** Total dose: 1E9 PFU	Day 1	5E8 PFU	intratumoral (via catheter)	15 min
	Day 10	5E8 PFU	intracerebral (direct injection at several locations in resection wall)	15–30 min

GROUP 2

Escalation level	Study day	Dose	Route of administration	Duration
**Level 1**[a] Total dose: 1E6 PFU	Days 1–5	1E5 PFU	intravenous infusion	2 h
	Day 10	5E5 PFU	intracerebral (direct injection at several locations in resection wall)	15–30 min
**Level 2** Total dose: 5E7 PFU	Days 1–5	0.5E7 PFU	intravenous infusion	2 h
	Day 10	2.5E7 PFU	intracerebral (direct injection at several locations in resection wall)	15–30 min
**Level 3** Total dose: 1E9 PFU	Days 1–5	1E8 PFU	intravenous infusion	2 h
	Day 10	5E8 PFU	intracerebral (direct injection at several locations in resection wall)	15–30 min

GROUP 3

Escalation level	Study day	Dose	Route of administration	Duration
**Level 4 (single level)** Total dose: 5E9 PFU	Day 1	2.5E9 PFU	intratumoral (via catheter)	15 min
	Day 10	2.5E9 PFU	intracerebral (direct injection at several locations in resection wall)	30–60 min

[a] Planned as a possibility, but not implemented.

Table S5. Schedule of trial procedures in patients of groups 1 and 3

Study procedure	Study visit (day)																													
	SC	1	2	3	4	5	6	7	8	9	10	11	12	13	14	15	16	17	18	19	20	21	22	23	24	25	26	27	28[A]	FU[B]
Written informed consent	•																													
Demography	•																													
HIV, HBV and HCV serology	•																													
Pregnancy test	•																													
Biopsy and histology [C]		•																												
Administration of ParvOryx [D]		•									•																			
Physical & neurological examination	•		•	•	•	•	•	•	•	•		•	•	•	•	•	•	•	•										•	•
Vital signs [E]	•		•	•	•	•	•	•	•	•		•	•	•	•	•	•	•	•										•	
12-lead ECG	•		•		•					•		•							•										•	
Cl. chemistry, haematology, clotting [F]	•		•		•		•			•									•										•	
Serum protein electrophoresis	•									•									•										•	
H-1 PV antibodies [G]	•	•	•	•	•	•	•	•	•	•	•	•	•	•	•	•	•	•	•	•	•	•	•	•	•	•	•	•	•	
MRI	•		•			•				•		•[H]																	•	•
Recording of adverse events		•	•	•	•	•	•	•	•	•	•	•	•	•	•	•	•	•	•	•	•	•	•	•	•	•	•	•	•	•
Shedding of H-1 PV [G]	•	•	•	•	•	•	•	•	•	•	•	•	•	•	•	•	•	•	•	•	•	•	•	•	•	•	•	•	•	
Tumour resection											•																			
Survival status and PFS																														•

SC: screening; FU: follow-up; Cl.: clinical.

A Flexible: day 26–30 or earlier, if viral shedding had ceased or there was seroconversion (see text).

B 1 month (± 7 days) after day 28, then every 2 months (± 14 days) up to 6 months after day 1.

C At discretion of responsible investigator, i.e. only if imperative for unequivocal confirmation of diagnosis.

D Intratumoral administration on day 1, intracerebral administration on day 10.

E Pulse rate, blood pressure, body temperature.

F Selected parameters.

G Discontinuation of monitoring depending on previously obtained results.

H Either on day 11 or on day 12.

Table S6. Schedule of trial procedures in patients of group 2

Study procedure	Study visit (day)																													
	SC	1	2	3	4	5	6	7	8	9	10	11	12	13	14	15	16	17	18	19	20	21	22	23	24	25	26	27	28[A]	FU[B]
Written informed consent	•																													
Demography	•																													
HIV, HBV and HCV serology	•																													
Pregnancy test	•																													
Biopsy and histology[C]		•																												
Administration of ParvOryx[D]		•	•	•	•	•					•																			
Physical & neurological examination	•		•	•	•	•	•	•	•	•		•	•	•	•	•	•	•	•										•	•
Vital signs[E]	•		•	•	•	•	•	•	•	•		•	•	•	•	•	•	•	•										•	
12-lead ECG	•		•		•					•		•							•										•	
Cl. chemistry, haematology, clotting[F]	•		•		•		•			•									•										•	
Serum protein electrophoresis	•									•									•										•	
H-1 PV antibodies[G]	•	•	•	•	•	•	•	•	•	•	•	•	•	•	•	•	•	•	•	•	•	•	•	•	•	•	•	•	•	
MRI	•		•			•				•		•[I]																	•	•
Adverse events		•	•	•	•	•	•	•	•	•	•	•	•	•	•	•	•	•	•	•	•	•	•	•	•	•	•	•	•	•
Shedding of H-1 PV[G,H]	•	•	•	•	•	•	•	•	•	•	•	•	•	•	•	•	•	•	•	•	•	•	•	•	•	•	•	•	•	
Tumour resection											•																			
Survival status and PFS																														•

SC: screening; FU: follow-up; Cl.: clinical.

A Flexible: day 26–30 or earlier, if viral shedding had ceased or there was seroconversion (see text).

B 1 month (±7 days) after day 28, then every 2 months (±14 days) up to 6 months after day 1.

C At discretion of responsible investigator, i.e. only if imperative for unequivocal confirmation of diagnosis.

D Intravenous administration on days 1–5, intracerebral/intratumoral administration on day 10

E Pulse rate, blood pressure, body temperature.

F Selected parameters.

G Discontinuation of monitoring depending on previously obtained results.

H On days 1–5, viremia was monitored by taking three blood samples per day from each subject at the following times: pre-dose, interval from 1 h after start of infusion to 1 h after stop of infusion, and interval from 2 to 6 h after end of infusion.

I Either on day 11 or on day 12.

Table S7. Sequence of locked nucleic acid (LNA) oligonucleotides used for FISH detection of H-1PV nucleic acids

**Sense probe**	
NS1	5DIGN/AATTCGCTAGGTTCAATGCGCT/3DIGN
VP	5DIGN/TGACCTACCAACATCAGATACA/3DIGN
**Antisense probe**	
NS1	5DIGN/TCAGCACACAACAGATGGCAT/3DIGN
VP	5DIGN/TACTATCCAGAGCAACCATCAT/3DIGN

The sense and antisense probes were synthesized so as to recognize the negative or positive strands of H-1PV nucleic acids, respectively, and double digoxin (DIGN) labeled at their 3′ and 5′ ends. NS1- and VP-specific probes were used as a mix of equal amounts, to increase the hybridization signal. Signals were visualized by incubation with anti-DIGN antibody conjugated to horseradish peroxidase (Roche, Sigma-Aldrich, Munich, Germany) followed by incubation with the Tyramide Signal Amplification/cyanine 3 reagent (Perkin Elmer, Rodgau, Germany). Images were acquired with a motorized widefield Cell Observer® microscope and the ZEN blue image-processing software (Carl Zeiss, Göttingen, Germany).

Table S8. Primary antibodies used for IF detection of tumor and tumor microenvironment markers

Marker	Primary antibody/Source	Provider
GFAP	ab7260/rabbit polyclonal	Abcam
EGFR	ab2430/rabbit polyclonal	Abcam
CD68	MO814/mouse monoclonal	DAKO
CTSB	ab125067/rabbit monoclonal	Abcam
CD45	ab8216/mouse monoclonal	Abcam
CD3	ab5690/rabbit polyclonal	Abcam
CD4	MA 1-80223/mouse monoclonal	Thermo Fisher Scientific
CD8	ab4055/rabbit polyclonal	Abcam
GZMB	ab139354/mouse monoclonal	Abcam
Perforin	ab75573/mouse monoclonal	Abcam
IFN-γ	ab9657/rabbit polyclonal	Abcam
IL-2	ab180780/rabbit polyclonal	Abcam
CD25	ab154393/rabbit polyclonal	Abcam
CD154 (CD40L)	sc-978/rabbit polyclonal	Santa Cruz
Iba1	ab15690/mouse monoclonal	Abcam
FOXP3	ab20034/mouse monoclonal	Abcam

Signals were visualized using cyanine 3/Oregon Green® 488-conjugated secondary antibodies. Images were acquired with a Cell Observer® microscope and the ZEN blue image-processing software (Carl Zeiss, Göttingen, Germany). GFAP, glial fibrillary acidic protein; EGFR, epidermal growth factor receptor; CTSB, cathepsin B; GZMB, granzyme B; IFN, interferon; IL, interleukin; Iba1, ionized calcium binding adaptor molecule 1; FOXP3, forkhead-box-protein P3.
